# Motor modules are largely unaffected by pathological walking biomechanics: a simulation study

**DOI:** 10.1186/s12984-025-01561-8

**Published:** 2025-01-30

**Authors:** Mohammad Rahimi Goloujeh, Jessica L. Allen

**Affiliations:** Department of Mechanical and Aerospace Engineering, University of Florida, PO Box 116250, Gainesville, FL 32611 USA

**Keywords:** Muscle coordination complexity, Gait analysis, Computer modeling, Optimal control, Trajectory optimization, Direct collocation

## Abstract

**Background:**

Motor module (a.k.a. muscle synergy) analysis has frequently been used to provide insight into changes in muscle coordination associated with declines in walking performance, to evaluate the effect of different rehabilitation interventions, and more recently, to control exoskeletons and prosthetic devices. However, it remains unclear whether changes in muscle coordination revealed via motor module analysis stem from abnormal walking biomechanics or neural control. This distinction has important implications for the use of motor module analysis for rehabilitation interventions and device design. Thus, this study aims to elucidate the extent to which motor modules emerge from pathological walking biomechanics, i.e. abnormal walking biomechanics commonly observed in individuals with neurological disease and/or injury.

**Methods:**

We conducted a series of computer simulations using OpenSim Moco to simulate pathological walking biomechanics by manipulating speed, asymmetry, and step width in a three-dimensional musculoskeletal model. We focused on these spatiotemporal metrics because they are commonly altered in individuals with Parkinson’s disease, stroke survivors, etc. and have been associated with changes in motor module number and structure. We extracted motor modules using nonnegative matrix factorization from the muscle activations from each simulation. We then examined how alterations in walking biomechanics influenced the number and structure of extracted motor modules and compared the findings to previous experimental studies.

**Results:**

The motor modules identified from our simulations were similar to those identified from previously published experiments of non-pathological walking. Moreover, our findings indicate that the same motor modules can be used to generate a range of pathological-like waking biomechanics by modulating their recruitment over the gait cycle. These results contrast with experimental studies in which pathological-like walking biomechanics are accompanied by a reduction in motor module number and alterations in their structure.

**Conclusions:**

This study highlights that pathological walking biomechanics do not necessarily require abnormal motor modules. In other words, changes in number and structure of motor modules can be a valuable indicator of alterations in neuromuscular control and may therefore be useful for guiding rehabilitation interventions and controlling exoskeletons and prosthetic devices in individuals with impaired walking function due to neurological disease or injury.

## Background

Motor module (a.k.a. muscle synergy) analysis has emerged over the last few decades as a useful method to characterize the complex coordination of the numerous muscles involved in movements such as walking. Motor modules reflect coordinated patterns of muscle activity that can be flexibly combined to meet the goals of different movement behaviors [1]. It is hypothesized that motor modules reflect an underlying nervous system strategy to overcome the complexity of controlling movement by grouping muscles into functional units. As such, many researchers are using motor modules to evaluate the effect of different rehabilitation interventions on neuromuscular control [2–12] and even to control exoskeletons and prosthetic devices [13–21]. However, since motor modules are identified from experimentally-recorded electromyography (EMG) data using numerical decomposition techniques such as principal component analysis or nonnegative matrix factorization [1, 22, 23], there is ongoing debate regarding whether they truly represent an underlying neural strategy or simply emerge from the biomechanics of the recorded movement. Incorporating EMG-derived motor modules into rehabilitation interventions and/or into controllers for exoskeletons and prosthetics introduces additional complexity compared to recording only movement biomechanics. If motor modules are simply emergent from movement biomechanics, then there may be no need to incorporate such complexity into these settings. Therefore, it is critical to understand to what extent EMG-derived motor modules reflect an underlying neural strategy to support their use in identifying neuromuscular deficits limiting walking function, guiding rehabilitation efforts, and facilitating control of exoskeleton and prosthetic devices.

Converging evidence suggests that recruiting a reduced number of motor modules to control walking contributes to impaired walking function. Several studies have identified that between four to six motor modules are needed to describe muscle activity in unimpaired walking [24–26]. Comparatively fewer motor modules are required to describe muscle activity in people with neurological deficits (e.g., stroke [7, 24, 27, 28] cerebral palsy [29] and Parkinson’s disease [30–32]) and musculoskeletal conditions such as osteoarthritis [33]. Given that each module in unimpaired walking is organized around producing biomechanical functions such as leg swing control and forward propulsion [34, 35], it is not surprising that individuals with a reduced number of motor modules typically walk with impaired walking function. For example, reduced motor module number in stroke survivors is associated with slower walking speeds and more asymmetrical steps [24, 27] and an inability to change speed, cadence, step length, and step height [28]. Moreover, increases in motor module number that occur with rehabilitation are associated with improved walking function [10, 36]. While the prevailing interpretation of these studies is that a decrease in motor module number causes impaired function, an alternative explanation is that the observed reduction in motor modules may be a consequence of the altered walking biomechanics rather than the cause.

Musculoskeletal modeling and simulation offer a means to disentangle the effects of neural control and biomechanics on motor modules in a carefully controlled manner. For example, Falisse et al., demonstrated a reduction in motor module number alone could not produce the crouch gait biomechanics often observed in cerebral palsy [37]. Moreover, Mehrabi et al., found that normal walking biomechanics could not be achieved with reduced motor module number [38]. Conversely, a recent experimental study provided some evidence that healthy individuals could emulate crouch gait patterns commonly observed in children with cerebral palsy without reducing motor module number [39]. Taken together, these studies suggest that although biomechanics may have some influence on motor modules, there remains room for them to have a neural basis. However, a limitation of these studies is their focus on a single gait type, preventing insights into the extent of biomechanics versus neural control on motor module structure across the diverse walking biomechanics often observed in populations with neurological disease and/or injury.

The purpose of this study was to explore the extent to which motor modules are emergent from pathological walking biomechanics. Or in other words, does pathological walking biomechanics *require* a reduced number and/or altered structure of motor modules. Inspired by the work of De Groote et al. [26] in which motor modules were extracted from individual muscle-driven simulations of unimpaired walking identical to how motor modules are identified experimentally from EMG, we compared motor modules extracted from 27 muscle-driven simulations of pathological gait biomechanics. We use the term “pathological walking biomechanics” to refer to measurable gait characteristics such as spatiotemporal parameters, kinematics, and kinetics resulting from injury or disease. In this study, we focused on the effect of varying speeds, step length asymmetry, and step widths commonly observed in populations with neurological disease or injury such as stroke survivors, persons with Parkinson’s disease, etc. We hypothesized that motor modules cannot be explained by biomechanics alone and thus reflect to some extent an underlying neural control strategy. Based on this hypothesis, we predicted that the number and structure of motor modules would not differ across simulations with different pathological walking biomechanics.

## Methods

A series of 27 different simulations were designed to model different pathological walking biomechanics encompassing both normal gait and those commonly encountered in populations with neurological disease and injury, including three speeds (0.8, 1.1, and 1.45 m/s), step length asymmetry levels (0, 15, and 30%), and step widths (0.1, 0.2, and 0.3 m). For each simulation, optimal muscle recruitment in a 3D musculoskeletal model over a single gait cycle was identified. To achieve this, the resultant optimal control problems were discretized through a direct collocation method solved by nonlinear programing techniques using OpenSim Moco [40]. Motor modules were then extracted from each of the 27 different optimal solutions from each leg for the (a) full set of 43 muscles per leg (full set) and (b) a reduced set of 8 muscles per leg similar to those from which EMG is typically collected experimentally (8-muscle subset). Finally, the number and structure of motor modules across different walking behaviors were compared to address our study hypotheses.

### Musculoskeletal model

The musculoskeletal model was a modified and armless version of the 3D OpenSim model originally described in Rajagopal et al. [41]. The modified model consisted of 14 rigid body segments representing the torso, pelvis, and both legs (femur, tibia, patella, calcaneus, talus, and toes) with a total mass of 73.5 kg and height of 1.75 m. The model had 23 degrees-of-freedom (DoF), with a 6 DoF pelvis as the root segment. The trunk and hip were both modeled with three rotational DoF. The knee was modeled as a pin joint with a single DoF. The ankle, subtalar, and toe joints were also modeled as single DoF joints. The model was controlled by 94 Hill-type musculoskeletal units using the OpenSim *DeGrooteFregly2016Muscle* model [42] (43 per leg and 4 on each side of the torso; Table 1). Three torque actuators were included around the torso joint to provide additional stability not provided by the trunk muscles. Passive torques representing forces applied by ligaments, passive tissues, and other joint structures were also added to all joints in the model via torsional spring-dampers according to the equations in [43, 44]. Ground contact was modeled using six Hunt-Crossley contact spheres on each foot: one of radius 3.5 cm positioned at the bottom of the heel, three of radius 1.5 cm at anterior portion of the calcaneus at toe joint, and two with the radius of 1.5 cm on toes. The stiffness and dissipation coefficients of the spheres were assigned 3.06 MPa and 2.0 s/m respectively to make the energy return similar to the heel region of the human foot in an athletic shoe [43].

**Table 1:** List of the muscles included in the model

Muscles	Muscles names
Int/Ex Obl	Internal/External Obliques
REAB	Rectus Abdominus
ERSP	Erector Spinae
Piri	Piriformis
Psoas	Psoas major
Sart	Sartorius
TFL	Tensor Fascia Latae
IL	Iliacus
AddB/L	Adductor Brevis/Longus
AddmI/D/M/P	Adductor Magnus Ischium/Distial/Middle/Proximal
GMAX1/2/3	Gluteus Maximus superior/middle/inferior
GMED1/2/3	Gluteus Medius anterior/middle/posterior
GMIN1/2/3	Gluteus Minimus anterior/middle/posterior
SM	Semimembranosus
ST	Semitendinosus
BFlh/sh	Bicep femoris long head/short head
GRAC	Gracilis
VM/I/L	Vastus medius/intermedius/lateralis
RF	Rectus femoris
EXd/h	Extensor digitorum/hallucis longus
PERb/l/t	Peroneus brevis/longus/tertius
TA	Tibialis anterior
FDb/l	Flexor digitorum brevis/longus
FHl	Flexor hallucis longus
TP	Tibialis posterior
FDb	Flexor digitorum brevis
GM/L	Gastrocnemius medial/lateral head
SOL	Soleus
POP	Popliteus

### Optimal control problem and objective function

Each of the 27 different desired walking behaviors was generated via discretizing the optimal control problem using the direct collocation method carried out in OpenSim Moco [40]. Each stride cycle was discretized into a total of 101 grid points adopting Hermite-Simpson transcription and the discretized problem was turned into a generic nonlinear programming problem using CasADi. This problem took the general form of:

1 $$\begin{array}{*{20}l} {{\text{Minimize}}} \hfill & {w_{effort} J_{effort} + w_{Tracking} (J_{ref} + J_{GRF} ) + w_{torso} J_{torso} + w_{asymmetry} J_{asymmetry} } \hfill \\ {{\text{subject}}\;{\text{to}}} \hfill & {{\text{multibody}}\;{\text{dynamics}}} \hfill \\ {} \hfill & {{\text{muscle}}\;{\text{model}}\;{\text{dynamics}}} \hfill \\ {} \hfill & {{\text{kinematic}}\;{\text{constraints}}} \hfill \\ {} \hfill & {{\text{prescribed}}\;{\text{average}}\;{\text{speed}}} \hfill \\ {} \hfill & {{\text{step}}\;{\text{width}}} \hfill \\ {} \hfill & {{\text{periodicity}}\;{\text{of}}\;{\text{the}}\;{\text{states}}} \hfill \\ {} \hfill & {{\text{initial}}\;{\text{and}}\;{\text{final}}\;{\text{states}}\;{\text{and}}\;{\text{controls}}} \hfill \\ {{\text{with}}\;{\text{respect}}\;{\text{to}}} \hfill & {{\text{initial}}\;{\text{and}}\;{\text{final}}\;{\text{time}}} \hfill \\ {} \hfill & {{\text{limits}}\;{\text{of}}\;{\text{state}}\;{\text{and}}\;{\text{control}}\;{\text{variables}}} \hfill \\ \end{array}$$

where w’s are the weights of each term and each J refers to a cost term to be minimized that is described in more detail in the below sections. In the sections below, we first focus on the three main spatiotemporal parameters of interest in this study: step length asymmetry (Sec. "Step length asymmetry goal (Jasymmetry)"), walking speed (Sect. "Speed constraint"), and step width (Sec. "Step width constraint"). Desired step length asymmetries were achieved via the objective function (J_asymmetrty_) whereas desired speeds and step widths were achieved via constraints. Following the presentation of these spatiotemporal parameters are descriptions of how other goals and constraints were modeled.

#### Step length asymmetry goal (J_asymmetry_)

The step length asymmetry term was modeled using the *MocoStepLengthAsymmetryGoal* and was part of the objective function to be minimized. Step length asymmetry was computed as (RSL-LSL)/(RSL + LSL), where RSL and LSL are right and left step length, respectively. Positive values correspond to larger right step lengths. This goal requires the target asymmetry and total stride length to be prescribed. Simulations of non-asymmetric walking did not include this term and the target asymmetry value was set to 0.15 and 0.30 to achieve 15% and 30% step length asymmetry. The total stride length depended on walking speed and was set to 1.04, 1.22 and 1.4 for 0.8, 1.1, and 1.45 m/s, respectively. These values were identified from the optimal symmetric walking simulations at each speed.

#### Speed constraint

Desired walking speed was achieved via the *MocoAverageSpeedGoal*. Use of this goal requires a desired value for the average speed of the center of mass and is implemented as a constraint such that the difference between desired and actual walking speed is zero, calculated as:

2 $$v_{des} - \frac{{r_{com} (t_{f} ) - r_{com} (t_{i} )}}{{t_{f} - t_{i} }}$$

where v_des_ is the desirable speed and the r_com_ is the center of mass position, and t_i_ and t_f_ represent initial time and final time.

#### Step width constraint

Step width was modeled as a distance constraint between the right and left foot (calcaneus and toe segments) via the *MocoFrameDistanceConstraint*. This constraint was utilized to keep the minimum sagittal-plane distance between the right and left foot segments greater than a prescribed value. The minimum distance between the feet in normal walking was 0.1 m and the step width constraint was used to produce wider steps of 0.2 m and 0.3 m.

#### Effort term (J_effort_)

Minimizing effort was modeled as minimizing the sum of the squared control signals (either muscle excitation or torque) over the entire simulation via the MocoControlGoal:

3 $$J_{effort} = \int_{{t_{i} }}^{{t_{f} }} {\sum\limits_{c \in C} {w_{c} } } \left| {x_{c} (t)} \right|^{2} dt$$

where x_c_ represents each control signal including the 94 MTUs and 3 torque actuators and w_c_ their respective weights, which were set equal to one.

#### Tracking terms (J_Ref_, JGRF, and J_torso_)

To achieve a realistic walking motion, three different tracking terms were included to track (1) reference kinematics, (2) reference ground reaction forces, and (3) an upright torso orientation.

1. Kinematic tracking was implemented using the *MocoStateTrackingGoal*, which minimizes the error between simulated joint angles and their reference trajectories (i.e., experimental data). The experimentally measured data [45] used as the reference kinematics included all joint angles except for the three lumbar rotations as well as the six positional and rotational values of pelvis (20 DOFs). The cost term was defined as [43]:
  4 $$J_{ref} = \int_{{t_{i} }}^{{t_{f} }} {\sum\limits_{i = 1}^{N = 20} {w_{i} (x_{i} (t) - \mu_{i} (t))^{2} } } dt$$
  where x_i_ is the value of i^th^ degree of freedom and µ_i_ is the average experimental value across subjects at time point *t*, and N is the number of DoF being tracked (23 DoF minus 3 trunk angles). The individual tracking weight for each joint was:
  5 $$w_{i} = \frac{1}{{N_{total} \times \sigma_{i}^{2} }}$$
  where $\sigma_{i}$ is the standard deviation of experimental data across all subjects of the ith joint averaged over the gait cycle and N_total_ = 29 (23 plus 3 components of ground reaction forces per leg).
2. Ground reaction force tracking was implemented using the *MocoContactTrackingGoal*, which minimizes the error between simulated ground reaction forces and their reference trajectories from [45]. The cost term was defined as:
  6 $$J_{GRF} = \int_{{t_{i} }}^{{t_{f} }} {\sum\limits_{i = 1}^{N = 6} {w_{GRF} (x_{i} (t) - \mu_{i} (t))^{2} } } dt$$
  where *i* refers to i^th^ force component including vertical, anterior-posterior and medial-lateral for both legs. The weight associated with each component was:
  7 $$w_{GRF} = \frac{1}{{N_{total} \times g \times m \times \sigma_{GRF}^{2} }}$$
  where g is gravitational acceleration, m mass of the model, $\sigma_{GRF}$ is the standard deviation of ground reaction forces across subject from the experimental data at time point *t*.
3. Upright torso orientation was implemented using the *MocoOrientationTrackingGoal*, which minimized the error between the three lumbar rotational DoFs and an upright torso orientation. The cost terms was defined as:
  8 $$J_{torso} = \int_{{t_{i} }}^{{t_{f} }} {(x(t) - \vec{V}_{reference} )^{2} } dt$$
  and weight equal to:
  9 $$w_{torso} = \frac{3}{{N_{total} \times \sigma_{torso}^{2} }}$$
  where ${\sigma }_{torso}$ is standard deviation of between-subjects experimental data of torso orientation.

#### Periodicity and initial and final state and controls

A periodic gait cycle was produced via the *MocoPeriodicityGoal*, which imposes equality between initial and final variables for all joint angles, angular velocities and muscle excitation except for pelvis anterior–posterior translation. Furthermore, the initial and final time of the simulation were prescribed such that the whole stride cycle was achieved over a fixed duration. The stride time prescribed for each simulation depends on the walking speed and was calculated according to the relationship described in Ziegler et al. [46]. Stride times were prescribed as 1 s for walking at 1.45 m/s, 1.15 s for walking at 1.1 m/s, and 1.35 s for walking at 0.8 m/s.

### Initial guess strategy

Twenty-seven distinct walking patterns were created by systematic deviation from experimental data of walking at 1.45 m/s from [45]. First, a solution for walking at 1.45 m/s with 0.1 m step width and symmetric step length was generated with kinematic tracking from experimental data [45]. This solution served as the initial guess for the optimization of symmetric walking at 1.1 m/s, which was then used as an initial guess for the optimization of symmetric walking at 0.8 m/s. Once simulations of symmetric walking with 0.1 m step width were achieved at all 3 different speeds, these solutions were used as the kinematic reference data and as the respective initial guess to achieve simulations with 15% asymmetry for each speed, which then served as the initial guess to achieve the 30% asymmetry solution. Then a similar approach was used for generating the 0.2 m and 0.3 m step width simulations at each speed and asymmetry level by changing the distance constraint values between feet.

### Motor module extraction

Motor modules were identified from each leg for each of the 27 different simulations via nonnegative matrix factorization (NMF) [47]. NMF decomposes muscle activity into a reduced set of motor modules according to the following formula:

10 $$\left[ E \right] = \left[ W \right] \times \left[ C \right] + e$$

where E is muscle activation matrix of size *m* × *101,* which represents number of muscles on each leg and number of grid points respectively. Matrix W represents motor modules (i.e., the groups of co-active muscles) and is of size *m* × *n*, where *n* is the number of modules. Matrix C is composed of the activation patterns of each of the *n* motor modules and is of the size *n* × *101*. e is the error of motor module extraction.

### Motor module analysis

The following analyses were performed on (1) all muscles per leg and (2) from a reduced 8-muscle subset per leg. This approach enabled us to compare our findings with existing literature, primarily derived from experimental data. The full muscle set included all muscles except the torso muscles due to the inclusion of the torso actuators, resulting in 43 muscles per leg. In contrast, eight muscles per leg were included in the subset: tibialis anterior, soleus, medial gastrocnemius, vastus medialis, rectus femoris, medial hamstrings, lateral hamstrings, and gluteus medius [24, 26].

#### Motor module number

To test our prediction that motor module number does not change with different walking biomechanics, the number of motor modules for each of the 27 different simulated walking behaviors were chosen as follows. For each data matrix (i.e., the *m* × *101* E matrix for a single leg within a single simulation), one to 10 (8 for the subset) motor modules (W’s) were extracted. The goodness of fit between actual and reconstructed muscle activity was evaluated with the variability accounted for (VAF), defined as:

11 $$VAF = 1 - \frac{{\sum\limits_{i = 1}^{m} {\sum\limits_{j = 1}^{t} {(e{}_{i,j})^{2} } } }}{{\sum\limits_{i = 1}^{m} {\sum\limits_{j = 1}^{t} {(E{}_{i,j})^{2} } } }}$$

where *t* and *m* represent number of time points and number of muscles, *E* is muscle activation from the simulations, and *e* represents the error of motor module extraction for each muscle at each time point (EMG reconstructed – *E*).

We chose the number of motor modules, n, for each simulation such that they could account for at least 95% of the patterns of muscle activation, i.e., > 95% VAF. Recognizing that this threshold is an arbitrarily chosen value and there lacks a universally accepted VAF cut-off for choosing motor module number, we further investigated the effect of different thresholds by assessing motor module number with thresholds of 90 and 95%.

#### Motor module structure

To test our prediction that motor module structure does not change with different biomechanics, two different analyses were performed. For each analysis, the simulated walking of speed 1.1 m/s, step length asymmetry of 0%, and step width of 0.1 m were chosen as the reference solution.

1. The W’s from each solution were compared to those of the reference solution using Pearson’s linear correlation.
2. We also evaluated the ability of the Ws from the reference solution to explain muscle activation in the other simulations. The motor modules activation (Cs) reconstruction were carried out as a nonnegative least square problem
  12 $$\begin{gathered} \min ||W_{reference} \times C_{reconstruct} - M_{t\arg et} || \hfill \\ {\text{Subject}}\;{\text{to}}\quad C_{reconstruct} \ge 0 \hfill \\ \end{gathered}$$

This was then solved using a nonnegative optimizer. Once reconstructed, the goodness of the reconstruction was evaluated using VAF. Larger VAF values for the reconstruction indicate that the W’s from the reference solution are better able to explain the muscle recruitment from the other walking behaviors. Note that the Cs of each these solutions were compared to assess differences in recruitment activity (Details in Sect. "Motor module activation patterns").

In addition, the similarity between right and left leg motor module structure due was examined using Pearson’s linear correlation.

#### Motor module activation patterns

**Fig. 1:**
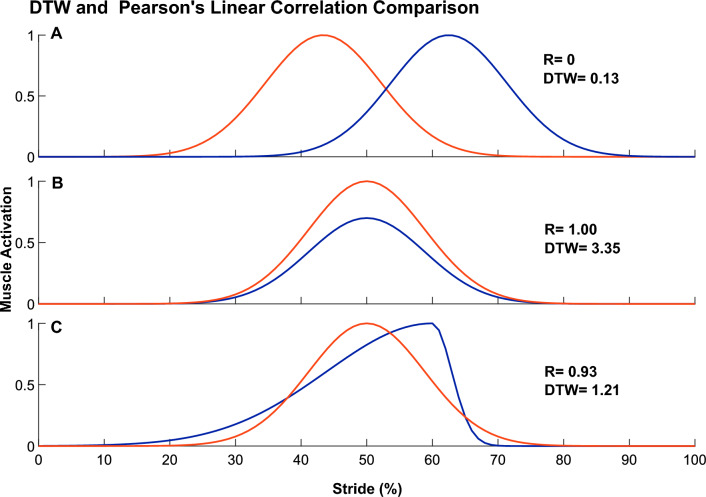
Comparison between pairs of two fake muscle activity patterns using dynamic time warping (DTW) compared to Pearson’s Linear Correlation Coefficients (R). Lower values of DTW indicate better match between curves whereas R close to 1 indicates better match between curves. **A** Identical muscle activities activated at different times result in a correlation of 0 (i.e., no match) but a DTW near zero (i.e., good match). **B** Identical muscle activities activated at different levels of magnitude result in a correlation of 1 (i.e., perfect match) but a relatively high DTW value (i.e., bad match). **C** Different muscle activity patterns activated with identical levels of magnitude and center of activity are also better captured using DTW

To explore how motor module activation patterns varied across different walking behaviors, the reconstructed Cs (as described above) were compared across simulations using three different outcome metrics:

1. Maximum activation to assess differences in peak module recruitment.
2. Center of activity (CoA) to assess differences in recruitment timing. CoA of each motor module activation was computed using circular statistics[48].
  13 $$\begin{gathered} A_{i} = \sum\limits_{t = 1}^{101} {(\cos \theta_{t} } \times C_{i,t} ) \hfill \\ B_{i} = \sum\limits_{t = 1}^{101} {(\sin \theta_{t} } \times C_{i,t} ) \hfill \\ CoA = \tan^{ - 1} ({\raise0.7ex\hbox{$B$} \!\mathord{\left/ {\vphantom {B A}}\right.\kern-0pt} \!\lower0.7ex\hbox{$A$}}) \hfill \\ where: \hfill \\ \theta_{t} = ({\raise0.7ex\hbox{${time_{t} }$} \!\mathord{\left/ {\vphantom {{time_{t} } {time_{f} }}}\right.\kern-0pt} \!\lower0.7ex\hbox{${time_{f} }$}}) \times 2\pi \hfill \\ \end{gathered}$$
  Here, *i* represents the *i*^th^ motor module activation for which the CoA calculation is carried out and *t* the time points. θ_t_ is the phase of the gait cycle which was derived by multiplying the normalized time vector and two radians accounting for the whole stride cycle.
3. Dynamic Time Warping (DTW) to assess similarity of each motor module’s activation pattern to that from the reference solution. DTW is a well-known algorithm to find the optimal alignment between timeseries and has found success in comparing biomechanical timeseries during gait such as ground reaction forces, muscle activations and joint angles [49–51]. DTW assesses similarities between two timeseries after aligning for temporal distortions, producing a measurement of similarity between motor module activation patterns irrespective of overall timing differences (e.g., heel-strike occurring at different timepoints). As a measure of distance between curves, lower values of DTW are associated with higher similarity. DTW is insensitive to timing of activity (Fig. 1A) but captures both shape (Fig. 1C) and maximum activation (Fig. 1B), and is thus a good complement to CoA. DTW analysis in this study was carried out using the DTW built-in MATLAB function.

## Results

### Target gaits

**Fig. 2:**
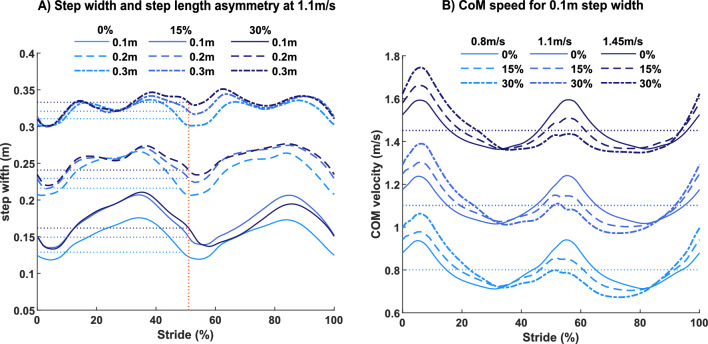
**A** Simulations of walking at 1.1 m/s with different step length asymmetry levels demonstrating the different step widths achieved. The minimum step width occurred around heel-strike (red dotted line) **B** Simulations of 0.1 m step width with different asymmetry levels demonstrating center-of-mass (CoM) speed over the gait cycle and its average (i.e., the target value for walking speed)

All 27 simulations converged to a stable solution. Constraints for step width (0.1, 0.2 and 0.3 m; Fig. 2A) and average walking speed (0.8, 1.1 and 1.45 m/s; Fig. 2B) were achieved and target asymmetry levels (0, 15 and 30%) were within 1% accuracy.

### Motor module number

**Fig. 3:**
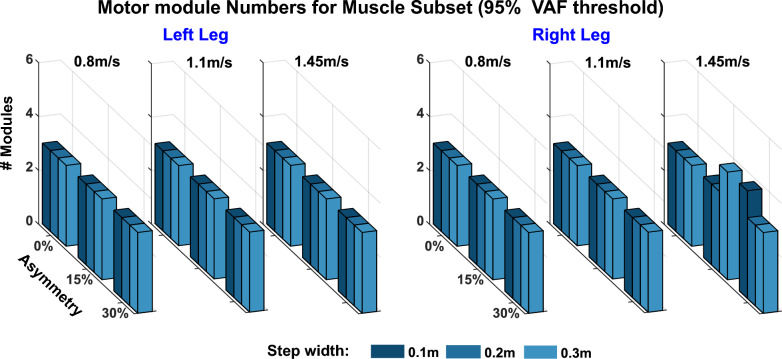
Number of motor modules to achieve 95% VAF for each simulation using the 8-muscle subset that includes muscles typically analyzed in experimental studies

**Fig. 4:**
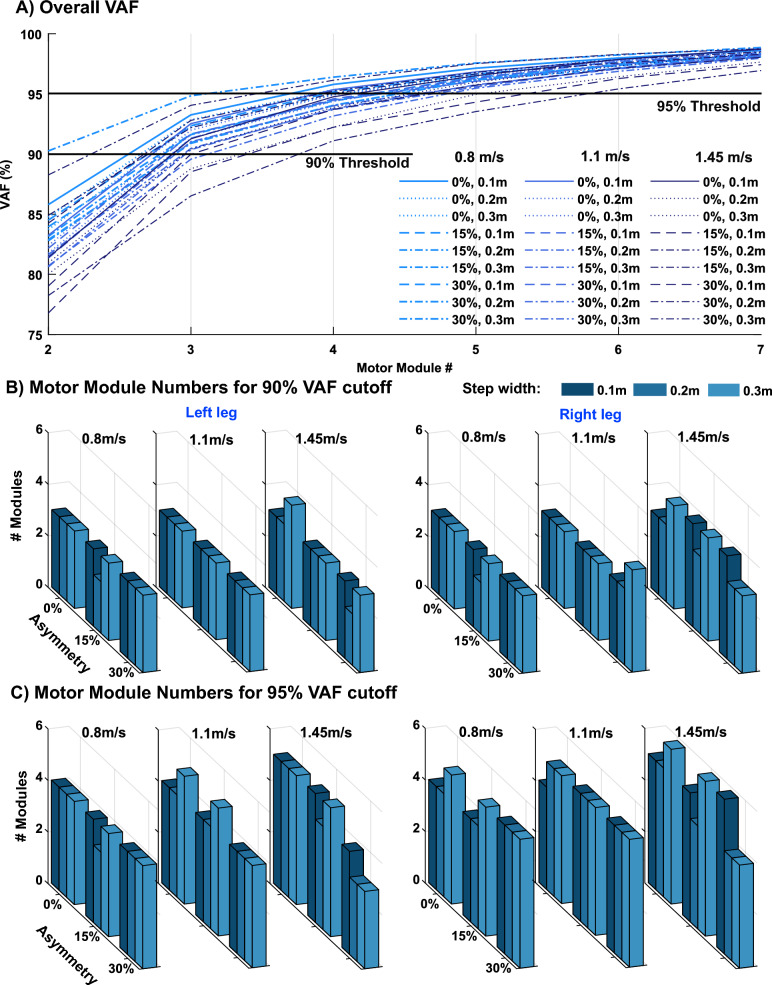
**A** Variability accounted for (VAF) in each simulation for each number of motor modules for all muscles on the right leg. **B** The number of motor modules required for each simulation to reach the 90% VAF threshold. **C** The number of motor modules required for each simulation to reach the 95% threshold

Motor module numbers were similar across simulations. When extracting motor modules from the 8-muscle subset with a 95% VAF cutoff, the number of motor modules was 3 for all walking behaviors in both legs (Fig. 3) except for the right leg at 1.45 m/s with 15% asymmetry combined with 0.2 m step width and 30% asymmetry combined with 0.1 m step width. Motor module number was increased and slightly more variable when extracting from the full muscle set. The median number of motor modules to achieve the 95% VAF threshold was 5 for both legs (Fig. 4B; left leg: range 3–5, 4.14 ± 0.59; right leg: range 4–6, 4.81 ± 0.61). There was a trend for an increase in motor module number with more complex gait biomechanics (i.e., faster walking speeds + more asymmetry + wider steps). However, the difference between whether 4 or 5 modules was needed came down to differences in VAF of 2% or less (Fig. 4A). When using the 90% VAF threshold to choose motor module number for the full muscle set, motor module numbers were more consistent across simulations with the median number of motor modules equal to 3 for both the left and right legs (Fig. 4C; left leg: range 2–4, 2.96 ± 0.33; right leg: range 2–4, 3.15 ± 0.45).

### Motor module structure

**Fig. 5:**
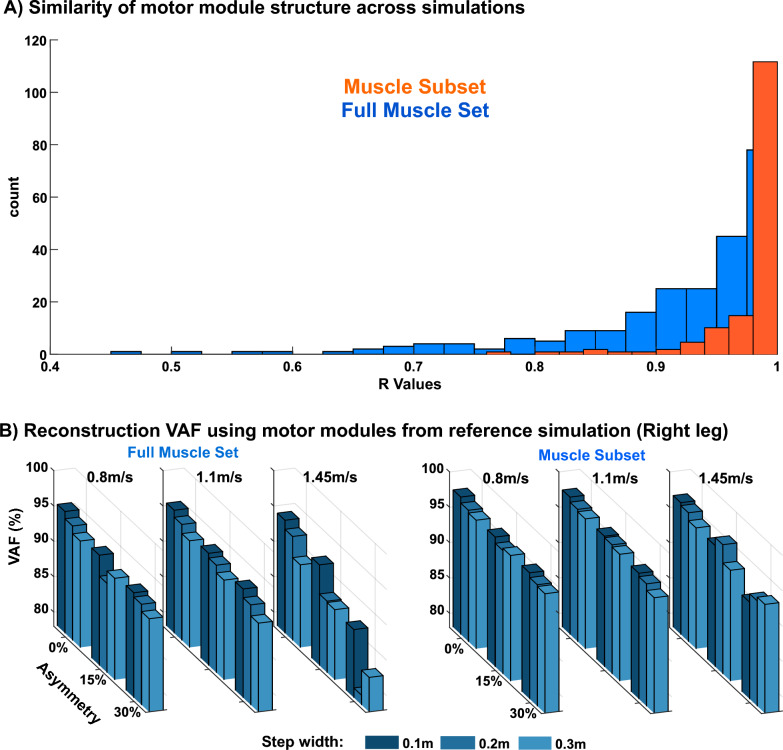
**A** Similarity in motor module structure when compared to those of the reference simulation for both the 8-muscle subset (orange) and full muscle set (blue) for both legs. **B** The variability accounted for (VAF) of muscle activity in the right leg when using motor modules from the reference simulation to reconstruct each of the other simulations

Motor modules with similar structure were recruited across the simulations with different biomechanics. Figure 5A shows a histogram of the similarity in motor module structure across all simulations in both legs. The correlation coefficient of the motor modules from each simulation compared to those from the reference solution (i.e., symmetric walking at 1.1 m/s with 0.1 m step width) extracted from the full muscle set was 0.91 ± 0.01 and 0.92 ± 0.08 for the right and the left leg, respectively. When extracting motor modules from the 8-muscle subset, the similarity in module structure was 0.98 ± 0.04 and 0.98 ± 0.02 for the right and the left leg, respectively.

The similarity of motor module structure across simulations with different walking biomechanics was confirmed when using the motor modules from the reference solution to reconstruct muscle activity in each of the other simulations (Fig. 5B). Reconstructed motor modules when looking at full muscle set explained muscle activity of all simulations with VAF of 0.91 ± 0.03 and 0.92 ± 0.02 for the right and left leg, respectively. The lowest VAF reconstruction of 79% was walking at 1.45 m/s with 0.2 m step width and 30% step length asymmetry, i.e., one of the most complex walking behaviors simulated. The reconstruction VAFs for 8-muscle subset were 0.95 ± 0.01 and0.96 ± 0.01 for the right and left leg, respectively.

**Fig. 6:**
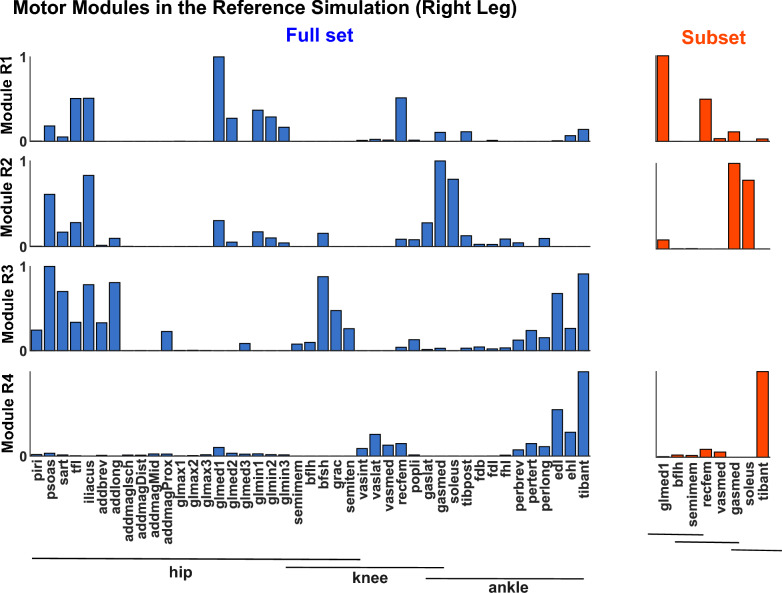
Motor modules on the right leg in the reference simulation (1.1 m/s, 0.1 m step width, and 0% step length asymmetry) for **A** the full muscle set and **B** the 8-muscle subset

**Fig. 7:**
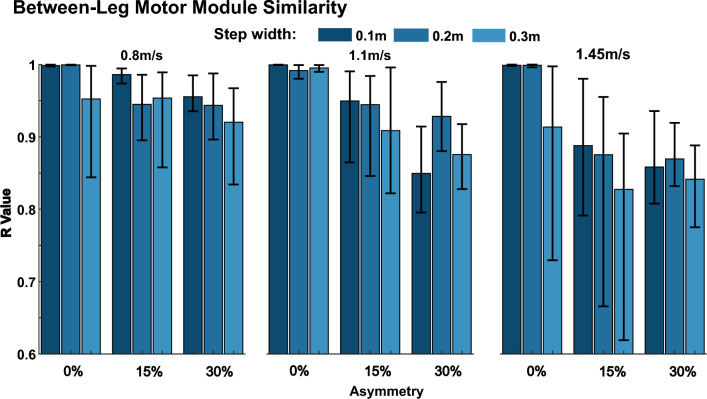
Similarity in motor module structure between legs in each simulation. The bars represent the average value of module comparisons and the error bars represent the minimum and maximum value of similarity

**Fig. 8:**
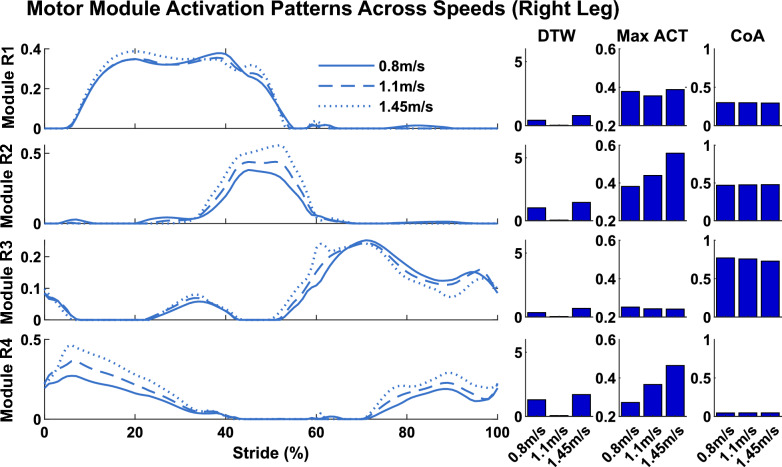
Comparison of motor module activation patterns across walking speeds. The solutions illustrated are those with 0% step length asymmetry and 0.1 m step width. DTW values were calculated in comparison to the reference simulation (i.e., walking at 1.1 m/s)

**Fig. 9:**
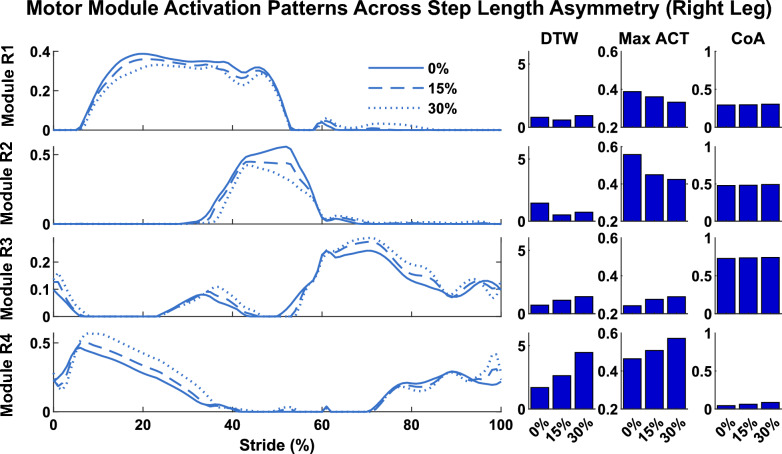
Comparison of motor module activation patterns across step length asymmetry. The solutions illustrated are those with walking speeds of 1.45 m/s and step width of 0.1 m. DTW values were calculated in comparison to the reference simulation (i.e., walking at 1.1 m/s)

**Fig. 10:**
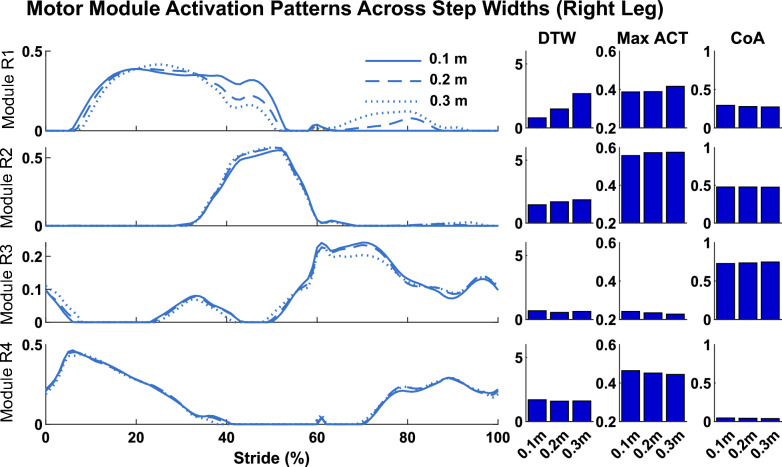
Comparison of motor module activation patterns across step widths. The solutions illustrated are those with walking speed of 1.45 m/s and symmetric step lengths. DTW values were calculated in comparison to the reference simulation (i.e., walking at 1.1 m/s)

Motor modules for the right leg of the reference simulation (1.1 m/s, 0.1 m step width, and 0% step length asymmetry) are illustrated in Fig. 6. Module R1 consisted of the glutei, knee extensors, and some hip flexors. This module was activated throughout much of the stance phase (Figs. 8, 9, 10). Module R2 consisted of the plantar flexors (gastrocnemius and soleus) with additional representation from the iliacus and psoas. This module was active during late stance. Module R3 consisted primarily of hip flexor muscles with additional activity from the hamstrings and ankle dorsiflexors. This module was active from late stance into swing. Module R4 consisted primarily of the ankle dorsiflexors and was active in late swing into early stance. The motor modules for the 8-muscle subset (Fig. 6B) were similar to those extracted from the full muscle set, with the exception that Module R3 was not identified due to, perhaps, the lack of hip flexor muscles in the experimental dataset. Motor module structure did not substantially differ between legs (Fig. 7), with most simulations resulting in modules over 85% similar between legs.

### Motor module activation patterns

Here, we compared the motor module activation patterns (i.e., the C’s) when using the reference solution motor modules (i.e., the W’s) to reconstruct muscle activity for each simulation. Peak activation and DTW values differed across solutions (Table 2, Figs. 8, 9, 10), but CoA remained largely unchanged. DTW values were calculated between the reconstructed C’s and the C’s from the reference solution (i.e., walking at 1.1 m/s with 0% asymmetry and 0.1 m step width). Specific differences in motor module activation patterns as a function of walking speed, step length asymmetry, and step width are presented in the below sections.

Motor module structure and timing comparison:

**Table 2:** Module module activation comparisons. DTW values represent the similarities and differences between reconstructed C’s and C’s of the reference simulations

Right Leg modules	Max activation	DTW	CoA
Module 1	0.37 ± 0.05	1.9 ± 1.46	0.29 ± 0.01
Module 2	0.44 ± 0.06	0.95 ± 0.44	0.48±0.01
Module 3	0.26 ± 0.03	1.18 ± 1.67	0.77 ± 0.01
Module 4	0.38 ± 0.1	2.02 ± 1.35	0.05 ± 0.01

#### Effect of walking speed

Motor module activation patterns changed with walking speed (Fig. 8; solutions for 0.1 m step width and 0% asymmetry across speeds). Module R1, which consisted of the glutei and knee extensors, exhibited changes in its activation pattern over the gait cycle as revealed by DTW. Qualitatively, these changes occurred due to peak activity levels extending longer in the stance phase, slightly altering the activation pattern shape. Module R2, which consisted of the plantarflexors, exhibited both increases in peak activation and changes in the activation pattern with walking speed. Qualitatively, the peak activity was reached earlier and maintained for longer when walking at faster speeds. Module R3, which consisted primarily of hip flexor muscles with additional activity from the hamstrings and ankle dorsiflexors, exhibited only small differences in activation patterns. Module R4, which consisted primarily of the ankle dorsiflexors, exhibited both increases in peak activation and changes in activation pattern with walking speed. Qualitatively, the peak activity was reached earlier in the swing phase and maintained for longer in the stance phase when walking at faster speeds.

#### Effect of step length asymmetry

Motor module activation patterns also changed with step length asymmetry (Fig. 9). The largest changes in activation patterns across different step length asymmetries occurred when walking at the fastest speed. Peak muscle activity increased in module R3 (hip flexors) and maintained heightened levels throughout swing, whereas modules R1 (glutei and knee extensors) and, especially, R2 (plantarflexors) exhibited decreased activity in late stance.

#### Effect of step width

Motor module activation patterns exhibited fewer changes across step width solutions (Fig. 10). The largest differences across different step widths occurred when walking at the fastest speed. Module R1 (which includes both hip flexor and abductor muscles) exhibited changes such that its activation was decreased in late stance and increased throughout swing phase. Recruitment of modules R2-R4 remained largely identical across step widths.

## Discussion

The purpose of this study was to explore the extent to which motor modules are emergent from pathological walking biomechanics versus representative of an underlying neural control strategy. Or in other words, do pathological walking biomechanics require a reduction in motor module number and/or alteration in their structures? Given the complexity of identifying motor modules compared to simply recording movement biomechanics, answering this question is critical to support the increasing use of motor modules to identify neuromuscular deficits limiting walking function, guide rehabilitation efforts, and control exoskeleton and prosthetic devices. To address this question, we utilized musculoskeletal modeling and simulation to evaluate the influence of various characteristics of pathological walking biomechanics on the number and structure of motor modules that explain muscle activity in the lower limbs. This approach allowed us to disentangle biomechanics from neural control, something that is difficult to do experimentally. We found that different walking speeds, step widths, and step length asymmetries could be achieved via the same motor modules. This supports our hypothesis that the differences in motor modules observed between individuals with and without pathological walking biomechanics (e.g., due to neurological disease or injury) reflect, to some extent, differences in underlying neural control strategies and not just differences in their biomechanics.

Motor module numbers were largely unaffected by different walking biomechanics. Three motor modules were required to explain simulated activity of the 8-muscle subset per leg in every simulation (Fig. 3). This number is consistent with prior studies that have identified between 3–5 motor modules in healthy adults from a similar set of muscles [24, 52–54]. Motor module numbers in the current study are likely on the lower end because (1) each simulation consisted of only a single gait cycle and (2) simulated muscle activity is less contaminated by noise than experimental EMG, both of which are known to lead to fewer motor modules [22, 55–59]. As expected and consistent with prior studies [60, 61], motor module numbers increased when extracting from the full set of 43 muscles per leg. Moreover, the consistency of motor module numbers across simulations was highly dependent on the criteria used to select motor module numbers. Motor module numbers were similar across simulations when using VAF cutoff of 90% but became slightly more variable across simulations when using VAF cutoff of 95%. Selecting the appropriate criteria for identifying motor module numbers has historically been a challenge, and it is well-recognized that these somewhat arbitrary cutoffs can impact results [22]. The increased variability in motor module numbers observed with a stricter selection criterion likely stemmed from reconstructing activity in muscles that play a minor role in locomotion, as muscle activity tends to be more variable when not substantially contributing to task demands [62–65]. As a secondary analysis of motor module numbers, we assessed how well the motor modules from the reference solution could reconstruct muscle activity from the other simulations. We found that over 80% VAF of the full muscle set could be explained in most simulations (Fig. 5B), confirming that the same number of motor modules could reconstruct muscle activity across simulations with various characteristics of pathological walking biomechanics. This value was even higher when looking at the 8-muscle subset with all the reconstructed VAF above 90%. These results contrast with previous experimental studies in which motor module number was reduced in those with pathological walking biomechanics (e.g., cerebral palsy, Parkinson’s disease, stroke, etc.) [7, 24, 27–32] and was in agreement with a previous study [39] in which similar motor modules were used by healthy individuals when emulating crouch knee gait patterns common in children with cerebral palsy. The findings of the current study provide further evidence that reductions in motor module number typically observed in populations with neurological disease and injury is not solely attributable to their altered walking biomechanics but also likely involves a neural component (e.g., neural injury, altered neural control strategy, etc.). Moreover, we extend the previous findings on crouch-like gait commonly observed in cerebral palsy [39] to altered spatiotemporal parameters commonly observed in individuals who have experienced a stroke or have Parkinson’s disease.

Motor module structure was also largely unaffected by different walking biomechanics. The motor modules extracted from the full muscle set in the reference simulation (i.e., “normal” walking biomechanics) (Fig. 6) were similar to those identified in other studies in which there were modules with dominant activity from: (1) hip/knee extensors in early stance, (2) ankle plantar flexors in late stance, (3) hip flexors and hamstrings from late stance into swing, and (4) ankle dorsiflexors in late swing into early stance [24–26, 66, 67]. Likely due to the absence of hip flexor musculature, motor module 3 was absent from the 8-muscle subset. We found that these same motor modules were present in all simulations regardless of walking biomechanics for both the full muscle set and the 8-muscle subset. The similarity of motor module structure across simulations was mostly greater than 0.9 for all comparisons of motor modules from the 8-muscle subset. Although the similarity was slightly reduced in the full muscle set, most comparisons remained above 0.85 similar, suggesting consistency in motor module structure across simulations (Fig. 5A). Moreover, that muscle activity could be reconstructed using the motor modules from the reference simulation (Fig. 6) with over 79% and 92% VAF in all simulations taking into account full set and 8-muscle subset respectively (Fig. 5B) confirmed the consistency of motor module structure across simulations. The different abnormal walking biomechanics were instead achieved via modulation of motor module activation patterns. How the pattern of recruitment of each motor module changed across conditions was consistent with their previously identified biomechanical roles [34, 35, 68]. For example, consistent with their respective role in body support and forward propulsion, modules R1 (hip/knee extensors) and R2 (plantarflexors) increased their activity in early and late stance as walking speed increased (Fig. 8). Consistent with their roles in controlling leg swing, module R3 (hip flexors) exhibited heightened activity with increasing step length asymmetry whereas module R1 (which also included hip adductor musculature) exhibited heightened activity with increasing step widths. Taken together, these results demonstrate that the same motor modules can be used to produce pathological-like walking biomechanics, providing evidence that alterations in motor module structure that occur in populations with neurological disease and injury have at least some neural origins.

While our study provides valuable insights into the role of biomechanics versus neural control in motor module structure and recruitment during walking, there are several limitations to consider. First, we investigated only a limited number of spatiotemporal characteristics of walking biomechanics: walking speed, step width, and step length asymmetry. This choice was motivated by the fact that these spatiotemporal characteristics are commonly altered in individuals with neurological disease/injury and accompanied by a reduction in motor module number [7, 24, 27–32]. Future studies should focus on other characteristics of pathological walking biomechanics. Second, our cost function included a term to track experimental kinematics from healthy walking to generate our initial simulations at each speed. However, the value of the weight assigned to this term allowed the solution still to deviate substantially from the tracked kinematics to achieve all desired abnormal spatiotemporal targets. Including the tracking term also helped to prevent unwanted changes in walking biomechanics other than the desired changes in speed, step length asymmetry, and step width. Thirdly, our cost function included a term to minimize the sum of squares of muscle activations as a way to minimize muscular effort. While it is generally agreed that minimizing effort is one of many goals during walking in both healthy and impaired populations [69–72], minimizing muscle activation may not represent the most accurate implementation of this goal. For example, prior simulation studies have found that minimizing the sum of squares of muscle activation does not always produce muscle activations consistent with experimental measurements of muscle activity via electromyography [73, 74]. In the current study, however, we were not concerned with mimicking muscle activation from a particular individual, but to generate realistic muscle activation patterns and comparing them across simulated walking behaviors. Moreover, the simulated motor modules were similar to previously published motor modules and were active at the right times in the gait cycle. While the specific makeup and timing of motor modules is likely to change slightly with different implementations of minimizing effort within the cost function, these minor changes would be unlikely to affect our study findings that the same motor modules can produce different walking biomechanics. Lastly, we used generic musculotendon parameters (e.g., muscle strength, tendon slack length, etc.) even though these parameters may change in individuals with neurological disease or injury. Prior simulation studies have shown that impaired musculotendon properties alone do not prevent normal walking biomechanics and that pathological walking biomechanics only emerged when combined with reduced motor module number and/or the goal to minimize energy expenditure [37, 75]. Therefore, we do not expect our main results demonstrating that pathological walking biomechanics can be achieved without a reduction in motor module number to be affected by this limitation. Future work should further explore the interaction between motor modules, musculotendon parameters, explicit models of altered neural control (e.g., spasticity), and optimality to identify what combinations of neural constraints and neural strategies lead to different types of pathological walking biomechanics.

## Conclusions

This study used predictive simulations to explore the role of pathological walking biomechanics on the number and structure of motor modules to explain lower limb muscle activity. We identified a consistent set of motor modules across simulations to muscle activity across varying walking speeds, step widths, and step length asymmetries. These results differ from experimental studies in which these same spatiotemporal walking biomechanics are associated with alterations in EMG-derived motor modules. Consequently, our results emphasize the significant impact of neural deficits and neural control strategies on alterations in EMG-derived motor modules. These insights provide support for the potential use of EMG-derived motor modules in identifying neuromuscular deficits, guiding rehabilitation interventions, and controlling exoskeletons and prosthetic devices in individuals with pathological walking biomechanics due to neurological disease or injury.

## Data Availability

All results and the codes necessary to replicate the simulations and extraction of motor modules are available at: https://github.com/Mohammad-Rahimi/different_walking_behaviors_directCollocation_NMF.

## Abbreviations

CoA: Center of activity
DTW: Dynamic time warping
DoF: Degree of freedom
EMG: Electromyography
MTU: Muscle–tendon unit
NMF: Nonnegative matrix factorization
VAF: Variability accounted for
